# Schulsozialarbeit in der „neuen Normalität“: Über neue und alte Herausforderungen in der Schulsozialarbeit, Entwicklungsmöglichkeiten und Chancen

**DOI:** 10.1007/s12054-020-00355-7

**Published:** 2020-12-30

**Authors:** Ingo S. Hettler

**Affiliations:** Duale Hochschule Baden-Württemberg (DHBW), Stuttgart, Deutschland

**Keywords:** Schulsozialarbeit, Soziale Arbeit an Schulen, Jugendhilfe und Schule, Covid 19, Corona-Pandemie, Lebensweltorientierung

## Abstract

Der mit der Covid-19 Pandemie verbundene Lockdown hat nahezu alle Bereiche des gesellschaftlichen Lebens vor große Herausforderungen gestellt. In Zeiten, in denen Schulen über Wochen hinweg geschlossen blieben, stellt sich in verschiedenen fachlichen und politischen Kontexten die Frage nach der Rolle der Schulsozialarbeit während des Lockdowns und in der „neuen Normalität“. Mit diesem Beitrag sollen verschiedene fachliche Überlegungen dazu angestellt werden, wie die Erfahrungen der Krise als Chance für eine stärkere Profilierung der Schulsozialarbeit genutzt werden können und sollten, und weshalb haushalterische Legitimationsdebatten an der Lebenswirklichkeit junger Menschen vorbeigehen.

Ausgehend von dokumentierten Erfahrungen aus Baden-Württemberg, die in zwölf Videokonferenzen durch Beteiligung von über 100 Schulsozialarbeiter_innen und über 50 Trägervertreter_innen in Baden-Württemberg zusammengetragen wurden, werden unterschiedliche Erfahrungen und Rahmenbedingungen während des ersten pandemiebedingten Lockdowns erläutert und Entwicklungspotentiale sowie Überlegungen für die Schulsozialarbeit in der „neuen Normalität“ und darüber hinaus aufgezeigt.

In dem weitreichenden Erfahrungsaustausch mit den Fachkräften der Schulsozialarbeit und Trägervertreter_innen in Baden-Württemberg wurde deutlich (Netzwerk Schulsozialarbeit Baden-Württemberg e. V. 2020), dass mit den Corona-bedingten Schulschließungen sehr unterschiedliche Konsequenzen für die Praxis der Schulsozialarbeit einhergingen, die auf unterschiedlichen Ebenen betrachtungswürdig sind. Eine Dimension bilden etwa die unterschiedlichen *materiell-technischen Ausstattungen* sowie die *trägerbezogenen *und die *kooperationsbezogenen Rahmenbedingungen* (Speck 2014, S. 100 ff.). So verfügte etwa nur ein geringer Teil der Fachkräfte über eine ausreichende berufliche IT-Infrastruktur, die eine – wenn auch reduzierte Erreichbarkeit – für die Adressat_innen ohne Rückgriff auf private Endgeräte und Telefonnummern überhaupt ermöglichte.

## Krise als bipolares Brennglas für bestehende Bedingungen und Herausforderungen

Während es an einigen Standorten gelang, institutionell organisierte Kommunikationsstrukturen aufrecht zu erhalten, in denen Fachkräfte der Schulsozialarbeit innerhalb des Trägers gemeinsame Ideen mit Kolleg_innen und ihren Vorgesetzten dafür entwickeln konnten, welche Prioritäten in der Krise gesetzt werden, wie Zugangswege zu den Adressat_innen kreativ gestaltet werden können und wie die Bedürfnisse und Bedarfe der Zielgruppe im Fokus behalten werden können, schienen viele andere Kollegen_innen sich selbst überlassen, d. h. ohne Austausch und ohne eine entsprechende fachliche Orientierung durch den Träger oder ein Schulsozialarbeitsteam zu sein. Dementsprechend ist es nur wenig überraschend, dass sich die Tätigkeitsschwerpunkte der Schulsozialarbeit regional auch während des ersten Lockdowns stark unterschieden. Die Bandbreite der Tätigkeiten in der Krise erstreckt sich dabei von Hintergrundarbeiten (z. B. Dokumentation, konzeptionelle Arbeit), über die Fortführung bereits begonnener Arbeitsprozesse und eine Fokussierung auf Notlagen in der Einzelhilfe (z. B. Kindeswohlgefährdung, familiäre Krisen) bis hin zur Entwicklung kollaborativer Strategien mit weiteren Partnern aus Jugendhilfe und Schule. Im Sinne eines „think outside the box“ wurde vielerorts deutlich, wie hilfreich und nützlich tragfähige Kooperations- und Netzwerkstrukturen im Sozialraum sind, um auch in Krisenzeiten funktionierende Wege der Kontaktaufnahme zu erschließen. Insgesamt wirkte die Krise für die Schulsozialarbeit gewissermaßen als bipolares Brennglas, in der sich die Wirkungen bereits bestehender Herausforderungen ebenso potenzierten wie die Wirkungen positiver Gelingensbedingungen. Deutlich wurde dieser Brennglaseffekt auch an der unterschiedlichen Kooperationsqualität mit Lehrkräften und Schulleitungen, die sich in der Krise bewährte oder in der die Zusammenarbeit der beiden Professionen mehr oder weniger zum Erliegen kam. (Netzwerk Schulsozialarbeit Baden-Württemberg e. V. 2020).

## Alter Wein in neuen Schläuchen?

Neben der konkreten Praxis sieht sich die Schulsozialarbeit mit erneuten Legitimationsfragen konfrontiert. Diese Fragen sind kommunal in sehr unterschiedliche Gewänder gekleidet, etwa in einem „Wohin“ als Form der Überprüfung und möglichen Aktualisierung der bisherigen Zielsetzungen von Schulsozialarbeit, oder auch einem „Wozu“, in dem die Weiterfinanzierung des Arbeitsfeldes – sicher auch vor dem Hintergrund haushalterischer Zwänge – grundsätzlich in Frage gestellt wird. In den Kommunen, in denen die Fragen nach dem „Wozu“ diskutiert werden, Verträge mit Trägern „vorsichtshalber“ zum Jahresende hin gekündigt werden und Einsparungsphantasien im Hinblick auf die Schulsozialarbeit zunehmend laut geäußert werden, gilt es auch deren Erwartungen an Schulsozialarbeit und ihre zugrundliegenden Begründungsmuster kritisch zu betrachten. Wird mit Schulsozialarbeit die Idee verbunden, junge Menschen „in ihrer Identitäts- und Persönlichkeitsentwicklung zu begleiten, in ihrer schulischen und außerschulischen Lebensbewältigung zu unterstützen sowie in ihren sozialen Kompetenzen zu fördern“ (Speck 2014, S. 54), so bedarf es auch in der „neuen Normalität“ einer bedarfsgerechten Einrichtung und Bereitstellung von Schulsozialarbeit für alle Schüler_innen.

Wer Schulsozialarbeit hingegen mit Erwartungen überlädt als „Allheilmittel zur Lösung persönlicher, schulischer, familiärer und gesellschaftlicher Problemlagen“ (Speck und Olk 2014, S. 38) zweckentfremdet, der wird zwangsläufig feststellen müssen, dass Schulsozialarbeit diese nicht erfüllen kann. Es stellt sich daher die Frage, ob die neuen Legitimationsdiskurse vielerorts nicht vielmehr altem Wein in neuen Schläuchen gleichen. Wenn die Schulsozialarbeit hingegen als wichtiger Bestandteil einer kommunalen Jugendhilfeinfrastruktur definiert wird und realistische Wirkungserwartungen an diese adressiert werden, dann können die Erfahrungen in der Coronapandemie auch als Katalysator für dringende Weiterentwicklungsbedarfe in einer Praxis der „neuen Normalität“ und darüber hinaus genutzt werden. Hierbei gilt es auch zu würdigen, dass die Schulsozialarbeit als „flexible situative Krisenmanagerin“ (Berndt et al. 2020, S. 20) – trotz ihrer zum Teil prekären Rahmenbedingungen (Berndt et al. 2020; Netzwerkschulsozialarbeit Baden-Württemberg e. V. 2020) – dazu beizutragen hat, stabile und arbeitsfähige Strukturen, insbesondere während den Schulschließungen, aufrechtzuerhalten.

## Schulsozialarbeit in der „neuen Normalität“

Mehrere aktuelle Studien und Stellungnahmen von unterschiedlichen kindheits- und jugendbezogenen Disziplinen und Expertenkreisen (Langmeyer et al. 2020; Andresen et al. 2020; DGPs und GEBF 2020; Fels et al. 2020; Fischer et al. 2020; Peter et al. 2020) weisen deutlich auf die möglichen Folgen der Pandemie für die emotionale und kognitive Entwicklung und das Wohl von Kindern und Jugendlichen hin. So fühlen sich nicht wenige junge Menschen mit den Herausforderungen des Homeschoolings, angespannten häuslichen Situationen und fehlenden Kontakten zu Freunden ohnmächtig und überfordert (Andresen et al. 2020, S. 9ff.). All diese Erfahrungen brachten die jungen Menschen nach der Wiederaufnahme des Schulbetriebes in ihren individuell bepackten Rucksäcken mit in die Schulen. Schule und Jugendhilfe sind dann gleichermaßen gefragt, junge Menschen bei der Bearbeitung dieser Erfahrungen zu begleiten und – wo nötig – weitergehende (sozial-)pädagogische Unterstützungsangebote vorzuhalten.

Für die Schulsozialarbeit bedeutet diese „neue Normalität“ jedoch nicht zwingend eine fachliche Neuausrichtung ihres Auftrages und ihrer Kernleistungen. Vielmehr gilt es, sich des originären Jugendhilfeauftrages und Charakters der Schulsozialarbeit zu erinnern und zu vergewissern. Mehr denn je scheint eine lebensweltorientierte Perspektive der Schulsozialarbeit gefragt zu sein, die sich anwaltschaftlich für die jungen Menschen einsetzt, ihnen eine Stimme schenkt und angemessen Gehör verschafft. Dies gilt gerade vor dem Hintergrund, dass sich über 40 % der in der bundesweiten JuCo-Studie befragten jungen Menschen „gar nicht“ oder „eher nicht“ gehört fühlten (Andresen et al. 2020, S. 11). Lebensweltorientierung in der Schulsozialarbeit bedeutet hierbei auch, den Fokus auf junge Menschen systematisch um deren digitalen lebensweltlichen Erfahrungen und Bezüge zu erweitern. Social-Media-Plattformen stellen inzwischen eine bedeutende Erweiterung jugendlicher Lebenswelten dar (Feierabend et al. 2019, S. 26 f.). Eine lebensweltorientierte Jugendhilfe sollte sich deshalb nicht verstecken hinter datenschutzrechtlichen Hürden und Herausforderungen, sondern sich „als parteiliche Vertretung lebensweltlicher Erfahrungen“ (Grunwald und Thiersch 2004, S. 23) in gegebene Macht- und Interessensstrukturen einmischen (ebd.). Für die Soziale Arbeit – auch außerhalb der Jugendhilfe – heißt dies, bundes- und europapolitisch darauf hinzuwirken, dass Social-Media-Plattformen dazu verpflichtet werden, den Schutz der persönlichen Daten der Adressat_innen DSGVO-konform sicherzustellen. Eine Erleichterung für die Praxis durch Lockerungen im Datenschutz kann dabei weder im Interesse der Sozialen Arbeit sein noch der Klient_innen und Zielgruppen, die sie vertritt. Die Schulsozialarbeit ist hierfür auch auf konkrete Unterstützung von Kommunen und Ländern angewiesen, die sich nicht länger passiv zum Status quo verhalten dürfen, indem sie strukturelle Probleme nach „unten“ durchreichen und damit zu individuellen Problemen und Entscheidungskonflikten von Schulsozialarbeiter_innen und deren Anstellungsträgern machen. Sie müssen sich ebenfalls engagieren und wo nötig für eine Verbesserung der Rahmenbedingungen und Verhältnisse einsetzen. Dies gilt auch für eine zeitgemäße Ausstattung der Arbeitsplätze der Fachkräfte, die deren Anschlussfähigkeit (sowohl im systemischen als auch im informationstechnologischen Sinne) an digitale jugendkulturelle Entwicklungen und Lebenswelten junger Menschen überhaupt erst ermöglicht.

## Die institutionelle Trennung von Schulsozialarbeit und Schule hat sich bewährt

Die Organisation und Trägerschaft der Schulsozialarbeit werden bereits seit Beginn der ersten Modellversuche kontrovers diskutiert. Obwohl sich die Trägerformen in der Praxis vielfältiger darstellen, werden im fachlichen Diskurs meist drei Trägerformen gegenübergestellt: Eine Trägerschaft in schulischer Verantwortung und zwei Trägerschaftsmodelle in Verantwortung der Jugendhilfe (freier Träger und öffentlicher Träger) (Stüwe et al. 2015, S. 220). Jedes Trägermodell geht dabei mit spezifischen Vor- und Nachteilen einher (Speck 2006, S. 247 ff.). Im Hinblick auf die Erfahrungen in der Krise zeichnet sich ab, dass sich die dezentrale Organisation, die sozialräumliche Vernetzung innerhalb der Jugendhilfe vielerorts bewährt haben, um kreative Ideen und Konzepte zu entwickeln, mit denen junge Menschen auch noch während eines Lockdowns möglichst niedrigschwellig erreicht werden (Netzwerkschulsozialarbeit e. V. 2020). Es ist davon auszugehen, dass eine schulische Trägerschaft und ein für beide Bereiche zentralisiertes Krisenmanagement bei den Ländern, nicht die erforderlichen Rahmenbedingungen für den Auftrag der Schulsozialarbeit in der gebotenen Differenzierung zum Auftrag der Lehrkräfte zu realisieren vermag, gerade dann nicht, wenn die Erfüllung des schulischen Auftrags durch Maßnahmen des Infektionsschutzes grundsätzlich in Frage steht. Mit einer Trägerschaft der Jugendhilfe wird hingegen gewährleistet, dass die Situation der Schüler_innen aus zwei unterschiedlichen Handlungsrationalitäten heraus Beachtung findet.

## Entwicklungsnotwendigkeiten

Nach wie vor fehlt es der Schulsozialarbeit bundesweit an einer rechtlichen Regelung, die der faktischen Bedeutung der Schulsozialarbeit gerecht wird. De jure erbringt Schulsozialarbeit damit ihre Leistungen gegenwärtig im rechtsfreien Raum (Kunkel 2016, S. 36). Aus dem Beteiligungsprozess zur Novellierung des SGB VIII ist derzeit nicht davon auszugehen, dass der Bund dieses Problem mit der kommenden Aktualisierung auflösen wird. Es wäre sehr bedauernswert, wenn diese Chance wiederholt vertan werden würde. Eine rechtliche und fachliche Absicherung der Schulsozialarbeit in der Kinder- und Jugendhilfe erscheint obligatorisch, um die Schulsozialarbeit mit ihrem sozialpädagogischen (Bildungs‑)Auftrag (Thiersch 2009, S. 28) und spezifischen Mehrwehrt für junge Menschen, deren Familien, den Sozialraum und die Schule zu stärken. Die Jugendhilfe ist dabei aber auch gefragt, entsprechende fachliche Supportstrukturen vorzuhalten, die „eine fortwährende Selbstvergewisserung und ‚Re-Kalibrierung‘ der Fachkräfte im Hinblick auf ihre professionelle sozialpädagogische Identität“ (Hettler 2020, S. 418) fördern. Nur so kann sich die Schulsozialarbeit langfristig hin zu einer Kooperation auf Augenhöhe emanzipieren, wenn ihre fachliche Expertise und ihr sozialpädagogischer Handlungsspielraum keine schulbedingte Engführung (mehr) erfährt. Zugleich muss sich Schulsozialarbeit immer wieder auf Schule zubewegen, wenn sie zukünftige Entwicklungen vor Ort anwaltschaftlich im Sinne der Kinder und Jugendlichen und unter sozialpädagogischen Gesichtspunkten mitgestalten möchte. Dies schafft Schulsozialarbeit keinesfalls allein. Sie braucht Verbündete, die ihre Zielsetzungen in ausreichendem Maße teilen und zwar bei Lehrkräften, Schulleitungen und schulischen Verantwortungsträger_innen. Dieses komplexe Zusammenspiel von „Abgrenzung von“ und „Zugehen auf“ Schule geht dabei auch einher mit einem besonderen Mehrwert für die Jugendhilfe selbst, da sie über Schule Gruppen von jungen Menschen erreicht, die ihr sonst verborgen bleiben würden.

Neben dem Bundesgesetzgeber sind aber auch die Länder und die Kommunen gefragt:

- Die Länder, indem sie die Gelingensbedingungen für die Kooperation von Schule und Schulsozialarbeit in den Schulgesetzen berücksichtigen und
- die Kommunen, indem sie bei der Finanzierung von Schulsozialarbeit auch Rahmenbedingungen mitdenken, die es den Fachkräften der Schulsozialarbeit ermöglicht, junge Menschen auch in ihren digitalen Lebenswelten abzuholen. Hier den Anschluss an die Schüler_innen zu finden war auch vor dem Corona-bedingten Lockdown schon ein Feld, das es vielerorts noch zu bestellen galt.

Fragen nach der Rolle und Aufgaben der Schulsozialarbeit zeichneten sich bereits während des Lockdowns ab. So wurden Schulsozialarbeiter_innen teilweise in fremden Bereichen eingesetzt, um beispielsweise die Notbetreuung in der Schule sicherzustellen. Zwar scheinen sich bestimmte Prioritäten nach den zahlreichen Wochen im Krisenmodus verändert zu haben, hiervon sollte sich die Schulsozialarbeit jedoch nicht in ihrem Selbstverständnis und Auftrag verunsichern lassen. Vielmehr sollte sie sich in ihrer Rückbesinnung auf die Lebensweltorientierung bestätigt fühlen, deren Struktur- und Handlungsmaxime auch nach 30 Jahren nicht an Aktualität verloren haben, deren Verwirklichung jedoch an die aktuelle Zeit und im Hinblick auf digitale Räume hin neu interpretiert werden müssen.

Die sozialpädagogische Perspektive wird zur Bearbeitung der Erfahrungen während des Lockdowns und den damit verbunden Entwicklungsrisiken mehr denn je gebraucht – jedoch nicht, indem Schulsozialarbeiter_innen als hauptverantwortliche Krisenbeauftrage ihren Fokus ausschließlich auf Krisen und Kinderschutzfälle legen, sondern indem sich diese als Anwält_innen für die Bedürfnisse junger Menschen und deren vielfältigsten lebensweltlichen Erfahrungen und Bezügen verstehen. Schulsozialarbeit kann hierfür einen wertvollen Beitrag leisten, damit Jugendhilfe und Schule als starke Partner_innen einer Verantwortungsgemeinschaft zusammenwirken. Legitimationsdiskurse zur weiteren Finanzierung der Schulsozialarbeit wirken angesichts der deutlichen Expert_innenhinweise deplatziert und ungemessen. Einsparungen im Bereich der Schulsozialarbeit konterkarieren wichtige Potenziale der Kooperation von Jugendhilfe und Schule, die einer gemeinsamen Verantwortung und gemeinsamen Herausforderung im Hinblick auf die Bearbeitung der Folgen der Lockdowns und die Alltagsgestaltung in einer „neuen Normalität“ gerecht werden müssen.
